# Optimization of Mulberry Extract Foam-Mat Drying Process Parameters

**DOI:** 10.3390/molecules27238570

**Published:** 2022-12-05

**Authors:** Nguyen Minh Thuy, Vo Quoc Tien, Nguyen Ngoc Tuyen, Tran Ngoc Giau, Vo Quang Minh, Ngo Van Tai

**Affiliations:** 1 Institute of Food and Biotechnology, Can Tho University, Can Tho 900000, Vietnam; 2 College of Environment and Natural Resources, Can Tho University, Can Tho 900000, Vietnam; 3 School of Food Industry, King Mongkut’s Institute of Technology Ladkrabang, Bangkok 10520, Thailand

**Keywords:** mulberry, foaming agent, foam characteristics, drying foam mat, anthocyanin

## Abstract

Mulberry powder was created from the extract using a foam-mat drying process. The studies aimed to evaluate the effects of egg albumin, carboxymethyl cellulose (CMC), digestion-resistant maltodextrin (DRM) contents, and whipping time (5 to 15 min) on the foam properties. The impact of different drying temperatures (60 to 75 °C) on the quality of the finished mulberry powder was also noted. The best foam expansion/stability value was determined using multiple regression models as a function of egg albumin, CMC, DRM, and whipping time. The results indicated that the main influencing factors for the foam properties were whipping time followed by egg albumin, CMC, and DRM. Optimum values of foam expansion and stability were achieved at 467.9% and 97.02%, respectively. The foam had a porous structure and good stability for subsequent drying, with optimal contents of egg albumin, CMC, and DRM used at 7.6%, 0.4%, and 2%, respectively, along with a whipping time of 14.5 min. The established models had a high coefficient of determination (R^2^ > 0.9) and a high correlation between the predicted and observed values. Therefore, the model could be adjusted to determine the characteristics of the foam suitable for subsequent drying. The optimal values were then also verified. Minimal fluctuations (1.78–2.98%) between the experimental data and the optimal value were found. The drying temperature also significantly affected the quality of the mulberry powder. The foam was dried at 65 °C for 4 h to produce apowder with a beautiful light color (L* = 62.65), a characteristic purple-red color of mulberry (a* = 5.97). The moisture, water activity, and anthocyanin content of the finished mulberry powder were 4.57%, 0.3, and 5.4 mg/g, respectively.

## 1. Introduction

Mulberry (*Morus* sp.) has been cultivated worldwide for fruit, fodder, and other purposes in severalAsian countries [1]. Mulberry belongs to the Moraceae family, and the genus Morus grows well in various climates. It is a small deciduous tree that grows in a variety of tropical, subtropical, and temperate climates, including China, Japan, Korea, Thailand, Indonesia, India, Vietnam, Brazil, and Africa [2]. In Vietnam, mulberry is often grown on riverbanks, flatlands, and plateaus. The main farming areas are the Red River, Day River, Thai Binh River, and Lam Dong, scattered in the Mekong Delta. The leaves, roots, branches, and bark of mulberry fruit are frequently used in traditional remedies because of their antioxidant and health advantages [3]. In particular, this fruit contains a large amount of anthocyanin, a water-soluble biologically active part of the polyphenol class and a specific substance that gives mulberry fruit its color.

Different colors of mulberries, even of the same species, can be attributed to the different amounts of anthocyanin they contain. The mulberry fruit has been reported to have 23 times the anthocyanin level of grapes [4]. The principal anthocyanins extracted from mulberry fruit are cyanidin-3-rutinoside and cyanidin-3-glucoside [5]. Several studies have shown that anthocyanins in mulberry fruit have an antioxidant activity and free radical scavenging capabilities [6]. Furthermore, the anthocyanin component of mulberries has numerous health benefits, including a lower risk of coronary heart disease, strokes, certain types of cancer, and aging [7]. As they possess many promising properties for human health, ripe mulberries are increasingly being exploited in the food industry to produce value-added food products. However, mulberry fruit is perishable and sensitive to mechanical and fungal damage, particularly after the harvest [8]. Mulberry fruit is also only available seasonally; therefore, determining how to prepare and preserve it into practical goods is a common issue in the food sector. In addition to the applied product processing technologies, beautiful-colored mulberry fruit can also be used as a food coloring agent, so extraction is also carried out. However, light, pH, oxygen, temperature, and enzymes all have an impact on the stability of anthocyanins in extracts [9]. In addition, the exceptionally high water content in the fruit leads to a short shelf life and complex storage.

Drying is a significant process for preserving various fruit and vegetables. Drying technologies such as freeze drying, microwave drying, vacuum drying, and infrared drying are commonly used [10]. However, it requires high investment and energy costs, and sometimes a high quality of the products [10,11]. As a result, hot-air drying can be used, which is usually lower than the starting material [12]. However, this method has disadvantages as well, including excessive energy consumption and low sensory, nutritional, and functional qualities. Due to the shrinkage and texture compactness phenomena that take place during traditional hot-air drying, the effective moisture diffusivity is reduced. As the food is exposed to heat for an extended time during the drying process, the time it takes to lose water should be reduced to reduce the loss of product quality [13]. In order to overcome these drawbacks, foam-mat drying, which is a technique of foaming from a liquid, has the ability to alleviate this issue. The liquid is beaten to make a stable foam before being dried on a foam pad at low temperatures [14]. Foam-mat drying is appropriate for heat-sensitive, dense, and sticky materials that cannot be dried using other methods such as spray drying; mulberry juice is an example. The study of Kanha et al. [15] showed that the total anthocyanin content and encapsulation efficiency of foam-mat-dried powders were comparable with and higher than the anthocyanin powders of spray- and freeze-dried black rice bran, respectively. Many fruit and vegetables have been dried using foam mats, including mangoes [16,17], bananas [18], tomatoes [19], bael fruit powder [20], papaya [21], and magenta leaf extract [14]. However, the application of foam drying technology to mulberry extract has not been studied. Recently, the study of Farid et al. [11] reported that good foaming properties could maintain the antioxidant compound in the observed powder due to the effect of the drying time. A high correlation between the foaming properties and antioxidant compounds was also found [11].

Therefore, we focused on determining the factors affecting the foaming process (volume expansion and foam stability), improving the necessary parameters, and properly preparing for the drying process at the next stage. The powder obtained from the foam drying method should maintain a beautiful color and the highest anthocyanin content.

## 2. Results

### 2.1. Physicochemical Composition of Mulberry

Table 1 shows the results of the investigation of the content of a few of the physical and chemical features of mulberry. 

The anthocyanin content of mulberry was analyzed ata value of 6.36 mg/g. The pH value of mulberry was measured as 3.6. The color of the ripened mulberry was analyzed. It was observed that the measured L* and a* values were 69.54 and 18.63, respectively.

### 2.2. Foam Formation and Stability

Mulberry extract was obtained from the material at a water ratio of 1:3 (*w*/*w*) and contained an anthocyanin content of 166.63 ± 1.76 mg/L at 3 ± 0.2°Brix. The L* and a* values were 67.37 ± 1.9 and 20.36 ± 0.7, respectively. The extraction foaming process was tested with fixed parameters, including the ratio of an added carrier (6% albumin, 0.3% CMC, and 1.5% RDM (*w*/*w*)), and whipped for 10 min. The foam volume and foam stability were found to be 639.33 ± 3.06 mL and 95.41 ± 0.16%, respectively.

### 2.3. Effect of Albumin, CMC, Digestion-Resistant Maltodextrin, and Whipping Time on the Properties of the Foam System

#### 2.3.1. Foam Expansion

A multi-level factorial design was used to arrange the influencing parameters (egg albumin, CMC, DRM, and whipping times; 3 levels) on the dependent variables (expansion and foam stability). A multiple regression analysis was used to analyze the correlation of the independent and dependent variables. The combination of the factor levels that maximized the foam characteristics over the indicated region was shown. Table 2 shows that the correlation model had linear coefficients (A, B, C, and D), interaction coefficients (AB, AC, AD, BC, BD, and CD), and quadratic coefficients (A^2^, B^2^, C^2^, and D^2^).

In this case, a multiple regression model was the appropriate statistical model to predict the foam expansion. It could be seen that egg albumin (A), CMC (B), maltodextrin resistance (C), and whipping time (D) all significantly affected the foam expansion (*p* < 0.05). The albumin-squared interactions (A^2^) and whipping time (D^2^) as well as thedouble albumin interactions and whipping time (AD) also showed similar results (*p* < 0.05). The remaining interactions did not significantly affect the degree of foam expansion (*p* > 0.05). The *p*-value of the lack of fit was also used to evaluate the model fit. After removing the non-significant interactions in the ANOVA table, acorrelation model was established (Equation (1)). The standard error of the estimate was 33.67 and the standard deviation of the residuals was 33.67.

Foam expansion (%) = −572.99 + 151.20 A+ 167.9 B + 32.47 C + 43.01 D − 11.18 A^2^ + 1.41 AD − 1.78 D^2^ R^2^ = 0.9; R^2^ (adjusted for Df) = 0.9; SEE = 33.67 (1)

where A is egg albumin (%), B is CMC (%), C is DRM (%), and D is the whipping time (min). 

**Figure 1:**
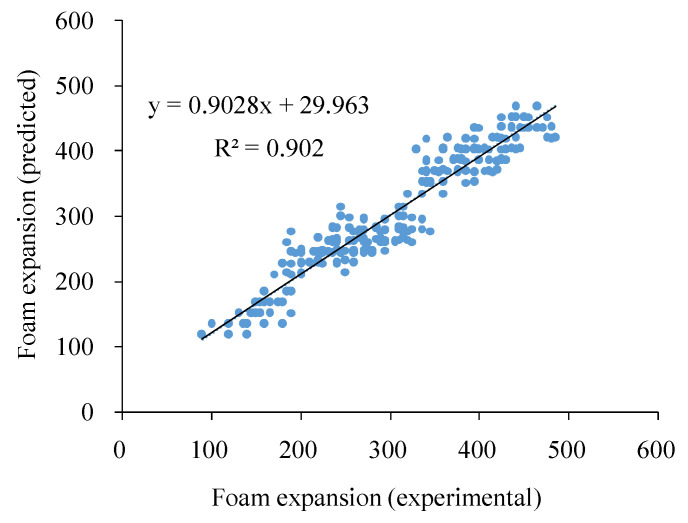
Correlation between experimental and estimated foam expansion values using the Equation (1) model.

By inserting the experimental values of the variables into Equation (1), the foam expansion could be predicted and the correlation between the observed and predicted foam expansion according to the equation was found (Figure 1) with a high correlation coefficient of determination (R^2^ = 0.90). 

To maximize the foam expansion, which was predicted to be a maximum of 468.67%, we used optimal egg albumin, CMC, and DRM values of 7.7%, 0.4%, and 2%, respectively, with a 15 min whipping time.

#### 2.3.2. Foam Stability (%)

The ANOVA table (Table 3) divided the changes in the foam stability into separate parts for each effect. In this scenario, *p*-values less than 0.05 indicated that 9 effects (A, B, C, D, A^2^, AD, BD, CD, and D^2^) were significant at the 95% confidence level. 

The model appeared to fit the observed data at the 95% confidence level because the *p*-value for the lack of fit in the ANOVA panel was larger than or equal to 0.05. According to the R^2^ statistics, the fitted model explained 90.73% of the variation in the foam stability. The corrected R-squared statistic as 90.16%, which was better for comparing the models with varied numbers of independent variables. The standard error of the estimate indicated that the standard deviation of the residuals was 0.95.

The regression equation was adjusted (after removing the non-significant interactions) to fit the data. Equation (2) of the fitted model was:

Foam Stability (%) = 52.06 + 5.93 A + 16.79 B + 2.01 C + 2.82 D − 0.35 A^2^ − 0.09 AD − 0.72 BD − 0.12 CD − 0.07 D^2^ (2)

where A is egg albumin (%), B is CMC (%), C is DRM (%), and D is the whipping time (min).

**Figure 2:**
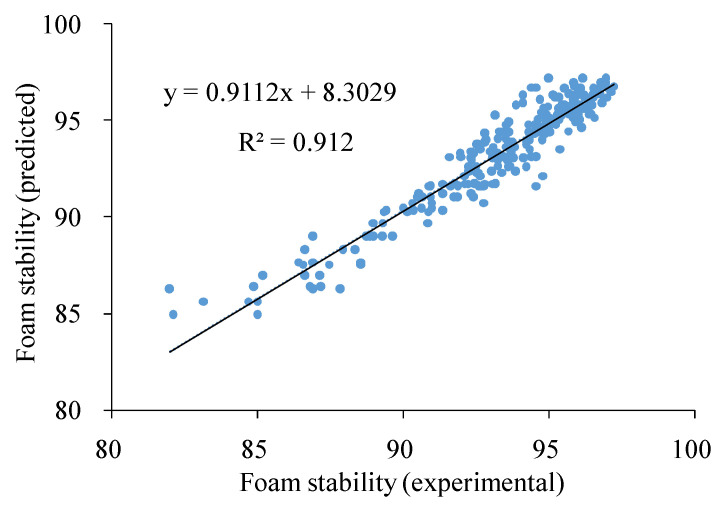
Correlation between experimental and estimated foam stability values using the Equation (2) model.

By inserting the experimental values of the variables into Equation (2), the foam stability could be predicted, and the correlation between the observed and predicted foam stability according to Equation (2) was found (Figure 2). The correlation coefficient of determination was relatively high (R^2^ = 0.91).

The factor level combination that enhanced the foam stability over the relevant region was presented. The best foam stability was anticipated at 97.62% with the addition of egg albumin, CMC, DRM, and a whipping time of 7%, 0.4%, 2%, and 12.25 min, respectively, for an optimal response.

#### 2.3.3. Optimized Desirability

Optimizing amulti-response surface is challenging to optimize all output responses together. The response surface methodology was used to individually optimize the dependent variablesin this study such as the foam expansion (%) and foam stability (%). As a result, somewhat different ideal values were achieved for the independent variables such as egg albumin, CMC, DRM, and whipping time. Desirability optimization should have yielded a mixture of response surfaces that maximizedthe foam expansion and stability whilst maintaining the same ideal egg albumin, CMC, DRM, and whipping time values. 

**Figure 3:**
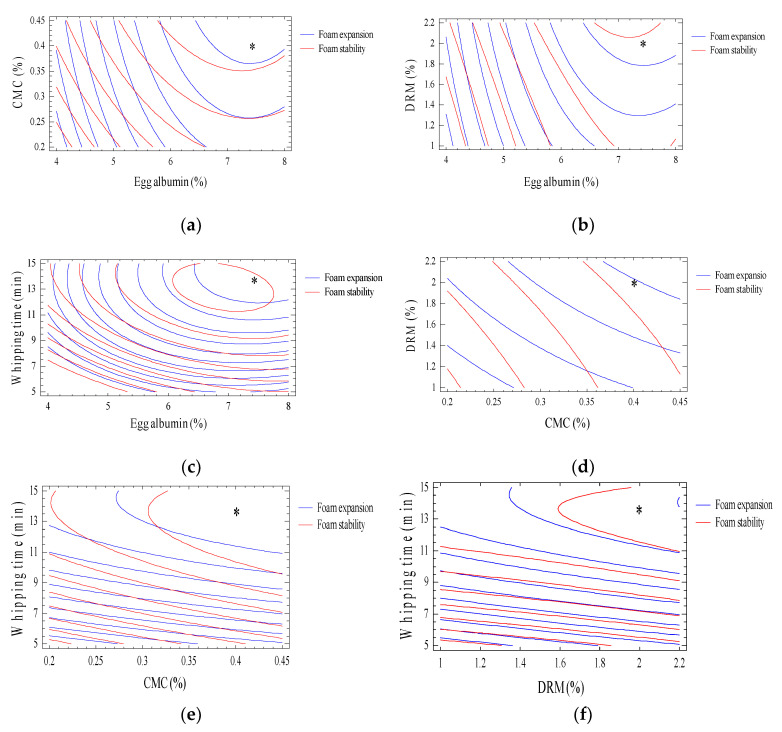
Overlay plot displaying the level of input variables and expected response values. (**a**) DRM 1.5%; whipping time 10 min. (**b**) CMC 0.3%; whipping time 10 min. (**c**) CMC 0.3%; DRM 1.5%. (**d**) Egg albumin 6%; whipping time 10 min. (**e**) Egg albumin 6%; DRM 1.5%. (**f**) Egg albumin 6%; CMC 0.3%. * is optimal point.

In Figure 3, a contour plot depicting the effect of egg albumin, CMC, DRM, and whipping duration on the optimal foam expansion (%) and foam stability (%) is highlighted by an asterisk (*).

The maximum values of the foam expansion and foam stability were 467.88% and 97.02%, respectively. The optimal values of egg albumin, CMC, and DRM were 7.6%, 0.4%, and 2%, respectively, and the optimal whipping time was 14.5 min. To test the optimal values found from the models, an experiment with a foaming process with optimal parameters (7.6% egg albumin, 0.4% CMC, 2% DRM, and 14.48 min of whipping time) was conducted to analyze the characteristics of the foam. The results showed that the foam expansion and stability were almost equivalent to the predicted results from the model. However, a very small discrepancy (1.75–2.98%) was discovered (Table 4), in which the foam expansion and foam stability were lower than the projected values of 2.98% and 1.75%, respectively. This difference was within the allowable limit (<5%).

### 2.4. The Effect of Drying Temperature on the Mulberry Powder Quality

Table 5 shows how the quality of mulberry powder changed with the drying temperature.

#### 2.4.1. Water Content and Water Activity

The results revealed that when the drying temperature increased from 60 to 75 °C, the moisture content of the raw materials fell progressively from 5.31% to 4.11%. The moisture level of the powder fell very slowly at 60 °C, and it took 5 h for the moisture content to drop to 5.31%. Meanwhile, it only took 4 h at drying temperatures of 65 and 70 °C for the powder to reach moisture contents of 4.57 and 4.3%, respectively. At a higher drying temperature (75 °C), although the product moisture decreased to 4.11% and took only 3.5 h, the product had a dark red-brown color mixed with a burnt smell. 

#### 2.4.2. Anthocyanin Content of the Mulberry Powder

When dried at 60 to 75 °C, the anthocyanin concentration of the mulberry powder ranged from 3.87 to 5.40 mg/g. In this study, a drying temperature of 65 °C was considered to be appropriate to maintain a high anthocyanin content and to retain the desirable properties of the mulberry pulp. Al-Farsi et al. [22] reported that when drying sour cherry powder at 65 °C, also obtained optimal results for theanthocyanin content.

#### 2.4.3. Color (L* and a*) of the Mulberry Powder

**Figure 4:**
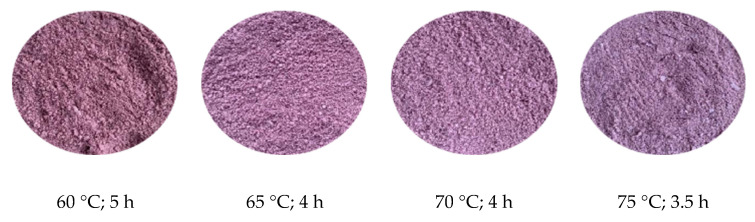
Mulberry powder color at various drying temperatures and durations.

It was observed that the samples dried at 60 °C for 5 h had a darker color than those dried at 65 and 70 °C for 4 h. The results showed that drying at 75 °C for 3.5 h madethe product darker. Samples dried at 60 °C had lower a* values (a* = 4.55 ± 0.35) than the other temperatures. The a* values of the samples dried at 65, 70, and 75 °C did not significantly change. The colors of the powder product at different drying temperatures areshown in Figure 4.

**Figure 5:**
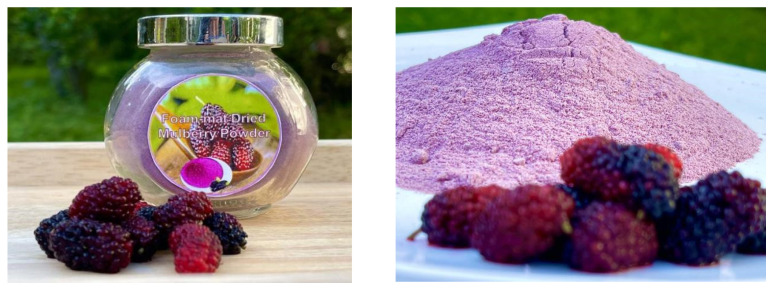
Mulberry powder obtained from the foam-mat drying technique.

It was observed that drying at 65 °C for 4 h maintained the characteristic color of mulberries (Figure 5).

## 3. Discussion

Fresh mulberries contain a relatively high water content. Our results were slightly higher than the published results of Imran et al. [23] and Yuan et al. [24]; the moisture content of mulberry was 81.72% and 87.68%, respectively.

The total soluble solid content (°Brix) of the mulberries in our study was lower than the analytical results of Kim et al. [4], who announced that the °Brix of mulberries grown in Korea was 14.6. 

Similar to the study by Lee et al. [25] on anthocyanin contents ranging from 0.39 to 9.25 mg/g indifferent mulberry varieties, Liang et al. [26] found a lower content of total anthocyanins in black mulberries, ranging from 0.19 to 1.93 mg/g FW. With a measured concentration of 5.67 mg/g, our achieved result was slightly higher than the data published by Farahani et al. [27].

The pH value of the mulberries in our study was quite similar to the study of Imran et al. [23]; the pH of mulberries in Pakistan was 3.35, but it was lower than the pH of mulberries grown in Turkey [28], with a published value of 5.6. The L* value was similar to the study of Ercisli and Orhan [28] on the Turkish mulberry (*Morus alba*) in Turkey (L* = 78.4), but was much higher than the study of Aramwit et al. [29] (L* = 27.72) for the Thai mulberry. In contrast, the analyzed a* values were similar to those reported by Aramwit et al. [29] (a* = 20.62) for the Thai mulberry, but higher than Ercisli and Orhan [28] (a* = −13.6) for the Turkish mulberry. The slight difference in the analytical results was probably due to differences in genetics, growing conditions, harvest times, and the ripeness of the mulberry fruit. Mulberry berries harvested when fully ripe usually have the best nutritional value [30]. Due to their high water content and thin skin, they should be consumed or processed as soon as possible because they are highly perishable [31]. From the analysis results, on the one hand, it was possible to confirm the quality of the input materials. However, on the other hand, it also showed that mulberry fruit contains a high water content, so it will easily spoil in tropical climates, especially the anthocyanin content. Therefore, foam drying could be an excellent way to maintain the desired values and obtain a suitable product with a long-term storage capacity.

As shown in the results, the foam volume and foam stability were found to be 639.33 ± 3.06 mL and 95.41 ± 0.16%, respectively. The lower and higher extraction ratios both produced a softer foam than the 1:3 extraction ratio (material:water) (data not given), mainly due to suitable juice viscosity that caused more foam to form; similar results were obtained when mandarin and papaya were foam-dried [20,21]. The foam stability is also important in foam-mat drying. The analysis showed only a minimal difference in the foam strength at different extraction rates (ranging from 94.98 to 95.75%). Kandasamy et al. [32] suggested that a reduced viscosity decreases the foam stability and increases the drainage volume. The higher the foam stability, the higher the foam quality. For speedy drying and the easy removal of the dried material from the tray, foam stability is also desirable. If the foam is fractured or overly drained, it takes longer to dry and the product quality suffers. Thus, the selection conditions were entirely suitable to determine the simultaneous change of the four factors for further studies.

The percentage of liquid that leaves the foam is determined by the foam stability, which shows the capacity of the foam to bind water [33]. In foam-mat drying, the foam stability is essential because it must be durable to retain its expansion structure during drying. 

Optimizing the multi-response surface is challenging to optimize all output responses together. As it is required for future industrial applications, research has been undertaken on the simultaneous optimization of several response surfaces [34,35,36]. The energy of the process decreases when the parameters are simultaneously optimized [37]. The response surface methodology was used to individually optimize the dependent variables such as the foam expansion (%) and foam stability (%) in this study. As a result, somewhat different ideal values were achieved for the independent variables such as egg albumin, CMC, DRM, and the whipping time. Desirability optimization should have yielded a mixture of response surfaces that maximized the foam expansion and stability whilst maintaining the same ideal egg albumin, CMC, DRM, and whipping time values. 

The foaming ability and foam stability are essential functions of egg albumin, which is why it is widely used in food processing. Foam is a colloidal system composed of small air bubbles spread in a continuous aqueous phase. The protein component in egg albumin acts as an amphoteric emulsifier between the air and water phases, so the foam is well-stabilized [38]. The level of foaming was low at low albumin concentrations; however, raising the concentration of egg albumin beyond the ideal point (>7.6%) did not result in substantial changes in the foam expansion. The albumin content used in this study was lower than that of the published literature. Thuy et al. [14] investigated the foam-mat drying of magenta leaf extracts and found that the optimum egg albumin and whipping time values were 12.21% and 5.8 min, respectively; their foam expansion values were lower (298.12%) than in our work. Affandi et al. [39] investigated the manufacturing of a *Nigella sativa* beverage powder and observed optimal foaming parameters of 15% egg albumen, 0.69% methylcellulose, and an 8 min whipping time. With these optimal parameters, the foam expansion ranged from 45% to 328%, and the foam stability ranged from 71% to 100%. Balasubramanian et al. [40] adjusted the process conditions for the creation of tomato foam with 11.45% egg albumin, 0.33% CMC, and a whipping duration of 5.21 min to achieve a 91.49% expansion volume and a 0.558 g/cc foam density.

The addition of CMC increases the viscosity of the surfactant solution and produces a stable foam with a smaller microbubble structure. Adding CMC at a concentration of0.4% did not affect the viscosity of the mixture at which the maximum air mass could be incorporated. The concentration of CMC recorded at 0.4% of the layout range showed the highest foam expansion and stability. Sangamithra et al. [41] reported that the optimum content of CMC used in muskmelon foam drying was 0.59%. 

High levels of resistant DRM were recorded (2%) to obtain the highest foam strength. At this concentration, the water-holding capacity increased and stabilized the foam system.

Whipping reflects the degree to which air is incorporated into the foam. If the aeration level is too high, the thinning of the liquid film between the foam bubbles as well as mechanical deformation might cause a rupture, increased water evaporation, and decreased foam stability. Our response surface models also showed that the foam stability tended to decrease with longer whipping times (longer than 14 min). Kandasamy et al. [21] also concluded that the foam formation and stability were maximal with a whipping time of 15 min at 1440 rpm at room temperature.

Moisture is the most significant property of food powders; it is usually less than 5%. A higher humidity can induce structural changes such as dough stickiness, caking, and other chemical changes. The consequences include a loss of product functionality and sensory values [42]. As shown in the results, the moisture content on the surface of the dried material evaporated faster than with low-temperature heating [43]. At a higher drying temperature (75 °C), although the product moisture decreased to 4.11% and took only 3.5 h, the product had a dark red-brown color mixed with a burnt smell. Franceschinis et al. [44] considered that aproduct with a 6% moisture content wassatisfactory in their research on blackberry powder. Along with adecrease in the moisture content, the water activity of powders tends to gradually diminish as the temperature rises. Ourdried powder product had a value from 0.243 to 0.324. It was observed that the drying performed at 65 °C was suitable for the mulberry powder to achieve a low water activity and avoid a color change compared with the original material; it also maintained a high anthocyanin content.

Anthocyanins are bioactive substances that degrade at high temperatures [45,46]. Therefore, maintaining the anthocyanin content in amulberry powder product is essential to maintain the quality of the powder. Similarly, Abbasi et al. [47], when drying sour cherry powder at 65 °C, also obtained optimal results for the anthocyanin content.

The key pigments responsible for color in mulberry fruit are anthocyanins, and the major chemicals found are cyanidin-3-glucoside and cyanidin-3-rutinoside [48]. This is similar to the research of Franco et al. [49], who, when studying yacon juice powder using foam drying technology, concluded that the brightness will decrease with a prolonged drying process. The L* value decreases, probably because increasing the drying temperature facilitates the reaction. The Maillard reaction and oxidation of anthocyanins can also give the product a dark color. Our acquired results were very comparable with the findings of Azizpour et al. [50], who discovered that increasing the drying temperature raised the a* value. The selection conditions for drying were 65 °C for 4 h, which maintained the characteristic color of the mulberries and created moisture and a_w_ suitable for product preservation. This result was quite similar to the study results of drying peach pulp [51] and sour cherry powder [47] at 65 °C.

The foam-mat drying method has been shown to be a simple, cost-effective technique with a fast drying time and well-maintained product quality. Based on the desirability of the early-stage foaming process variables, the foam formation and stabilization parameters were optimized at 7.6% egg albumin, 0.4% CMC, 2% DRM, and a whipping time of 14.5 min for achieving the maximum foam expansion and stability. In the next stage, the foam obtained from the mulberry extract was dried at 65 °C for 4 h and yielded a mulberry powder with a nice bright color that was well-preserved with a low moisture content (4.57 ± 0.01%) and water activity (0.30 ± 0.003). It could also be considered to be a suitable technology for producing food powders that retain highly biologically active compounds (anthocyanin in this case study). The initial data about the storability of the product showed that the product could be stored at 25 °C in a dark bag for a month with a very slight change in the nutritional quality. The obtained results are applicable to the food industry. The completed mulberry powder with exquisite hues can be used to generate attractive colors and substitute synthetic dyes in cakes, pasta goods, vermicelli, ice cream, and so on.

## 4. Materials and Methods

### 4.1. Sample Preparation

The mulberries were harvested in the An Giang province, Vietnam. First, they were selected according to their size and uniform ripening, with a red-violet color. The solid soluble content of the fruit was approximately 5 ± 0.5 °Brix (measured by a digital diffractometer, Tokyo, Japan). The mulberries were then washed, dried, and vacuum-packed; each bag weighed 5 kg. These were then frozen at −10 °C for further studies.

### 4.2. Foam Formation

The mulberry fruit was pureed with water at a ratio of 1:3 (*w*/*w*) using a 3-speed-level mixer (Philips HR 3705-300 W, Columbus, OH, USA) for 2 min at the highest speed. The pectinex enzyme was then introduced at a concentration of 0.03% (*v*/*w*) and incubated at 45 °C for approximately 30 min and the juice was recovered by vacuum filtration with a pore size of 20–25 μm. Finally, the foam formation and stabilization experiments were arranged with four factors. First, the mulberry extract was mixed with an emulsifier (egg albumin from 4 to 8%) and stabilizers (CMC from 0.2 to 0.4% and DRM from 1 to 2%) in the mixer (Philips HR 3705-300 W, Columbus, OH, USA) on the highest speed for 5 to 15 min for the foam formation.

### 4.3. Hot-Air Drying

The foam mixture was prepared from the selected optimum parameters of the emulsifier, stabilizer, and whipping time listed in Section 4.2. The mixture was transferred to stainless steel trays (1 m^2^) lined with parchment. The foam thickness of each tray was 4 mm. It was dried in an oven (MEMMERT, UN260, Bavaria, Germany) with an air velocity of 1.0 m/s at four different temperatures (60, 65, 70, and 75 °C) until the equilibrium moisture content (approximately 4%) of the sample was obtained. The powder was finely ground, collected after passing through a sieve with a pore diameter of 0.05 mm, and stored in dark conditions (4 °C) until the further analysis.

### 4.4. Foam Properties and Quality Analysis

#### 4.4.1. Foam Expansion

Using Equation (3), the foam expansion was estimated to determine the amount of air added into the solution during whipping [52].

(3) $$Foam\;expansion\left(\%\right)=\frac{V_{1}-V_{o}}{V_{o}}\times 100$$

where V_o_ is the initial volume of the mulberry extract (mL) and V_1_ is the volume of the extracted foam after the controlled whipping times (mL).

#### 4.4.2. Foam Stability

The whipped foam was placed in a transparent volumetric cylinder and left at room temperature (25 °C) for 2 h. The volume of the liquid removed from the foam by drainage was measured, and there was a decrease in the foam volume. The foam stability was computed using the description of Marinova et al. [53] (Equation (4)).

(4) $$Foam\;stability\left(\%\right)=\frac{V_{1}}{V_{o}}\times 100$$

where V_1_ is the foam volume after 2 h (mL) and V_o_ is the initial foam volume of the foam (mL).

#### 4.4.3. Total Anthocyanin Content (TAC)

The pH differential approach was used to calculate the TAC [54]. For measurements at 510 and 700 nm, a Cary 60 UV-Vis spectrophotometer (Agilent Technologies, Santa Clara, CA, USA) was used against a blank cell filled with distilled water. Equation (5) was used for TAC content (in terms of the cyanidin-3-glucoside equivalents):

(5) $$\mathrm{TAC}\left(\frac{\mathrm{mg}}{g}\right)=\frac{A\;\cdot {\;M}_{w}\cdot \;D\;\cdot \;F\;\cdot \;V\;\cdot \;1000}{a\;\cdot \;L\;\cdot \;m}$$

where A is the absorbance, M_W_ is the molecular cyanidin-3-glucoside weight (449.2 g/mol), DF is the dilution factor, V is the solvent volume (mL), a is the molar absorptivity (26,900$\;L\;\cdot \;{\mathrm{mol}}^{-1}\cdot \;{\mathrm{cm}}^{-1}$), and L is the cell path length (1 cm).

#### 4.4.4. Color

The color was measured according to L*, a*, and b* using a colorimeter (Tokyo, Japan).

#### 4.4.5. Water Activity

A water activity meter was used to measure the water activity of the foam-mat-dried mulberry powder at 25 °C (Aqua Lab Model Series 3TE, Decagon Devices, Inc., Pullman, WA, USA).

#### 4.4.6. Moisture Content

The moisture content of the raw material and product were determined by AOAC (2005).

### 4.5. Multiple Regression Analysis

The study used multiple regression to analyze the foam properties (volume expansion and foam stability). The model was fitted to the observed data using Statgraphics Centurion XVI software (USA). For each response (Y), a model (Equation (6)) was proposed:

(6) $$Y={\;b}_{o}+\overset{3}{\underset{n=1}{\sum }}b_{n}X_{n}+\overset{3}{\underset{n=1}{\sum }}b_{\mathrm{nn}}X_{n}^{2}+\overset{3}{\underset{n\neq m=1}{\sum }}b_{\mathrm{nm}}X_{n}X_{m}$$

where b_o_ is the Y intercept (constant), b_n_ is the regression coefficient for the linear effect of X_n_ on Y, b_nn,_ and b_nm_ are the regression coefficients for the quadratic effect on Y, and X_n_ and X_m_ are the independent values. The reference equation was chosen to fit the experimental data based on the R^2^ value obtained through the multiple regression.

## Figures and Tables

**Table 1:** A fewphysical and chemical characteristics of mulberry.

Items	Content
Moisture content (%)	89.7 * ± 0.9
Total soluble solid (°Brix)	5.00 ± 0.1
Anthocyanin (mg/g)	6.4 ± 0.4
pH	3.6 ± 0.3
Color	L* = 69.5 ± 0.6; a* = 18.6 ± 1.9

* Mean value ± STD.

**Table 2:** Analysis of variance for foam expansion.

Source	Sum of Squares	Df	Mean Square	F-Ratio	*p*-Value
A: Egg albumin	629,068	1	629,068	554.96	0.0000
B: CMC	45,669.1	1	45,669	40.29	0.0000
C: DRM	42,696.9	1	42,697	37.67	0.0000
D: Whipping time	1.03 × 10^6^	1	1.03 × 10^6^	906.21	0.0000
A^2^	108,004	1	108,004	95.28	0.0000
AB	39.12	1	39.12	0.03	0.8529
AC	1267.59	1	1267.59	1.12	0.2919
AD	21,533	1	21,533	19.00	0.0000
B^2^	266.67	1	266.67	0.24	0.6283
BC	144.68	1	144.68	0.13	0.7214
BD	2503.7	1	2503.7	2.21	0.1392
C^2^	66.67	1	66.67	0.06	0.8087
CD	2800.93	1	2800.93	2.47	0.1179
D^2^	106.67	1	106.67	94.10	0.0000
Lackoffit	22,440	66	340.01	0.30	1.0000
Pure error	183,633	162	1133.54		
Total (corr.)	2.2 × 10^6^	242			
R^2^ = 90.61%	R^2^ (adjusted for Df) = 90.03%			Standard error of est. = 33.67	

**Table 3:** Analysis of variance for foam stability.

Source	Sum of Squares	Df	Mean Square	F-Ratio	*p*-Value
A: Egg albumin	533.56	1	533.56	589.34	0.0000
B: CMC	148.30	1	148.30	163.81	0.0000
C: DRM	30.25	1	30.25	33.41	0.0000
D:Whipping time	1083.71	1	1083.71	1197.01	0.0000
A^2^	103.24	1	103.24	114.04	0.0000
AB	2.68	1	2.68	2.96	0.0875
AC	2.37	1	2.37	2.62	0.1076
AD	81.81	1	81.81	90.37	0.0000
B^2^	0.59	1	0.59	0.66	0.4189
BC	1.12	1	1.12	1.24	0.2676
BD	14.08	1	14.08	15.56	0.0001
C^2^	0.03	1	0.03	0.04	0.8490
CD	8.90	1	8.90	9.83	0.0020
D^2^	162.47	1	162.47	179.46	0.0000
Lackoffit	75.36	66	1.14	1.26	0.1215
Pure error	146.67	162	0.91		
Total (corr.)	2395.14	242			
R-squared = 90.73%		R-squared (adjusted for Df) = 90.16%		Standard error of est. = 0.95	

**Table 4:** The predicted and actual values of responses from ideal conditions.

	Predicted Value	Actual Value
Foam expansion (%)	467.88	453.94 ± 3.12
Foam stability (%)	97.02	95.3 ± 0.98

Mean value ± STD.

**Table 5:** Quality of mulberry powder at different drying temperatures and times.

Temp. (°C)	Time (Hour)	Moisture Content (%)	a_w_	Anthocyanin (mg/g)	Color	
					L*	a*
60	5	5.31 ± 0.22	0.324 ± 0.01	3.87 ± 0.13	59.93 ± 0.33	4.55 ± 0.35
65	4	4.57 ± 0.01	0.30 ± 0.003	5.40 ± 0.11	62.65 ± 0.69	5.97 ± 0.42
70	4	4.30 ± 0.26	0.29 ± 0.001	5.29 ± 0.12	64.13 ± 1.10	6.00 ± 0.33
75	3.5	4.11 ± 0.07	0.24 ± 0.001	4.66 ± 0.10	59.21 ± 0.86	5.95 ± 0.11

Mean value ± STD.

## Data Availability

Not applicable.

## Sample Availability

Samples of the extract from mulberry and powder are available from the authors.
